# Protein Expression of TXNIP in the Dopaminergic Neurons of Subjects with Parkinson’s Disease: Evidence from a Pilot Study

**DOI:** 10.3390/life15081252

**Published:** 2025-08-07

**Authors:** Francesca A. Schillaci, Giuseppe Lanza, Maria Grazia Salluzzo, Raffaele Ferri, Michele Salemi

**Affiliations:** 1 Oasi Research Institute-IRCCS, 94018 Troina, Italy; glanza@oasi.en.it (G.L.); msalluzzo@oasi.en.it (M.G.S.); rferri@oasi.en.it (R.F.); msalemi@oasi.en.it (M.S.); 2 Department of Surgery and Medical-Surgical Specialties, University of Catania, 95125 Catania, Italy

**Keywords:** Parkinson’s disease, TXNIP protein, immunohistochemistry, dopaminergic neurons, substantia nigra

## Abstract

Parkinson’s disease (PD) is a progressive, multisystemic α-synucleinopathy, recognized as the second most prevalent neurodegenerative disorder globally. Its neuropathology is characterized by the degeneration of dopaminergic neurons, particularly in the substantia nigra pars compacta (SNpc), and the intraneuronal accumulation of α-synuclein-forming Lewy bodies. Oxidative stress is a key contributor to PD pathogenesis. Thioredoxin-interacting protein (TXNIP) is a crucial regulator of cellular redox balance, inhibiting the antioxidant function of thioredoxin. This pilot study aimed to investigate the protein expression and localization of TXNIP in the SNpc of PD patients compared to healthy controls. We performed immunohistochemical analyses on 12 post-mortem human brain sections (formalin-fixed, paraffin-embedded) from six subjects with PD and six healthy controls. The study was performed on PD subjects with Braak stage 6. Our findings revealed that in control samples, TXNIP protein was distinctly and closely associated with neuromelanin (NM) pigment within the cytoplasm of SNpc dopaminergic neurons. Conversely, in PD samples, there was a markedly weak cytoplasmic expression of TXNIP, and critically, this association with NM pigment was absent. Furthermore, PD samples exhibited a significant reduction in both dopaminergic neurons and NM content, consistent with advanced disease. These findings, which mirror previous transcriptomic data showing TXNIP gene under-expression in the same subjects, suggest that altered TXNIP expression and localization in SNpc dopaminergic neurons are features of late-stage PD, potentially reflecting neuronal dysfunction and loss.

## 1. Introduction

Neurodegenerative diseases (NDs) are among the most prevalent diseases globally, particularly in middle- and high-income countries [1,2]. NDs are characterized by protein accumulation and selective anatomical vulnerability. Currently, their diagnosis relies on clinical and instrumental features, with definitive confirmation often only possible through post-mortem neuropathological evaluation [3,4]. Consequently, fluid diagnostic biomarkers and molecular imaging are areas of continuous research [3]. The most common NDs include amyloidosis, tauopathies, α-synucleinopathies, and transactive response DNA-binding protein 43 kDa (TDP-43) proteinopathies [3]. Alzheimer’s disease (AD), a tauopathy, is the most globally prevalent, followed by Parkinson’s disease (PD), an α-synucleinopathy. Both represent major societal issues and global health priorities [1,2,5].

PD is a multisystem α-synucleinopathy. Its cardinal motor symptoms, which facilitate diagnosis, include rigidity, resting tremor, bradykinesia, and postural imbalance. Non-motor symptoms—such as constipation, orthostasis, sphincter dysfunction, insomnia, REM sleep behavior disorder, mood disorders, anosmia, cognitive disorders, and pain and sensory disorders—are often detected in patients several years before the onset of motor symptoms [1,6,7]. Pathophysiological features of PD include neuronal destruction (particularly the loss of dopaminergic neurons, degeneration of nerve fibers, and loss of synapses), primarily observed in the substantia nigra pars compacta (SNpc), and the accumulation of α-synuclein-forming Lewy bodies (LBs), especially in the midbrain. Consistent with its nature as a multisystem disease, there is widespread involvement of various other Central Nervous System (CNS) structures and peripheral tissues [2,6,8,9], with α-synuclein pathology also being detectable in different biological fluids [10].

Although the diagnosis of PD still remains basically based on clinical features, it should be acknowledged that significant overlaps between PD and other clinical entities often cause diagnostic delay and challenging differential diagnoses, such as Progressive Supranuclear Palsy—Parkinsonism Predominant (PSP-P) and Multiple System Atrophy—Parkinsonism Predominant (MSA-P), particularly in early disease stages [11]. PSP-P, observed in up to 35% of PSP cases, manifests with akinetic rigid parkinsonism that may include tremor, asymmetry, or even partial levodopa responsiveness [12]. The diagnostic complexity is compounded by some PSP-P variants, such as that with progressive gait freezing, which may exhibit a levodopa-resistant freezing of gait earlier than typically seen in PD [13]. The same complexity holds true for MSA-P: both PD and MSA-P present with akinetic rigid parkinsonism, including bradykinesia, rigidity, and postural instability, and may initially respond to levodopa in some MSA-P cases [14]. Non-motor symptoms, like autonomic dysfunction and sleep disturbances, further blur diagnostic boundaries and complicate the management of PD [15]. However, MSA-P progresses more rapidly than PD, shows earlier and more severe autonomic failure, and develops distinctive clinical and instrumental markers [16]. In this context, clinical red flags, imaging biomarkers, and genetic correlates are very helpful, yet suboptimal, tools for distinction [17,18,19].

Dopaminergic neurons in the SNpc produce dopamine, a neurotransmitter whose depletion leads to reduced nerve impulses and, consequently, a loss of control over body movements. Additionally, these dopaminergic neurons are typically strongly pigmented due to the presence of neuromelanin (NM), making them easily recognizable by dark staining [20,21]. In PD, a key pathophysiological correlate of clinical motor symptoms is the loss of dopaminergic neurons in the SNpc. Subsequently, another possible link was identified between the reduction of NM-containing neurons (in the locus coeruleus and the dorsal motor nucleus of the vagus nerve) and the presence of non-motor symptoms [20]. Fasano et al. (2003) observed that LBs are localized in the cytoplasm in close physical association with NM. Indeed, their studies in human brains found that α-synuclein redistributes relative to NM pigment in the early stages of PD and becomes trapped in NM granules [22].

The thioredoxin-interacting protein (*TXNIP*) gene encodes a protein that binds to thioredoxin, an important regulator of cellular redox signaling which protects cells from oxidative stress. Specifically, the TXNIP protein, by binding to thioredoxin, inhibits thioredoxin’s antioxidant function, resulting in the accumulation of reactive oxygen species (ROS) and cellular stress (https://www.ncbi.nlm.nih.gov/gene/10628, accessed on 9 May 2025). A multitude of evidence indicates that the molecular and cellular pathophysiology of PD involves mitochondrial dysfunction, oxidative and nitrosative stress, the accumulation of aberrant or misfolded proteins, dysfunction of the ubiquitin–proteasome system, neuroinflammation, and autophagy [23,24,25].

In this preliminary pilot study, mainly due to the limited sample size, we performed immunohistochemical analysis on 12 post-mortem brain sections from two groups: individuals with PD in Braak stage 6 and healthy control subjects. In this study, we analyzed TXNIP protein expression, building upon our 2024 publication that reported the gene expression profiles from the same cohort [26]. Considering the previously identified mRNAs, we focused on the TXNIP gene, which was among those found to be significantly under-expressed (adjusted *p*-Value [padj] ≤ 0.05 and absolute fold change [|FC|] ≥ 1.5). Specifically, TXNIP exhibited a fold change of −2.625.

## 2. Materials and Methods

### 2.1. Post-Mortem Brain Sections

Post-mortem human brain sections from subjects with PD and controls were used in this study. Each section, mounted on the slide, was 4 μm thick and embedded in formalin-fixed kerosene (FFPE), which has the advantage of being easily preserved [27].

Tissue sections were sourced from the Multiple Sclerosis and Parkinson’s Tissue Bank located at Imperial College London, Hammersmith Hospital Campus, Du Cane Road (London W12 0NN, UK). The study included 6 samples from individuals with PD, comprising 4 men and 2 women with a mean age of 79.50 ± 5.39 years, alongside 6 control (CTRL) samples, consisting of 1 man and 5 women, with a mean age of 78.17 ± 10.38 years. All samples of PD subjects were in Braak LB stage 6. Neuropathological staging, developed by Braak, allows differentiation between early, intermediate, and late stages of PD-related lesions. According to this theory, subjects in stage 6 present involvement of the first-order sensory associative areas to the premotor fields and, occasionally, even to the primary sensory and motor fields; in the temporal lobe, it invades the cortex and reaches up to the primary auditory area. In addition, limbic damage is assumed to be a factor leading to the following cognitive decline [28]. Table 1 shows data from the six PD subjects and the six CTRL subjects (age, sex, and clinical condition of the subject at the time of death) obtained from the Multiple Sclerosis and Parkinson’s Tissue Bank at Imperial College London.

The research was conducted in compliance with the ethical standards outlined in the 1964 Declaration of Helsinki and its subsequent revisions. The study protocol received approval from the Ethics Committee of the Oasi-IRCCS Research Institute in Troina, Italy on 4 October 2025 (approval code: CEL-IRCCS OASI/10-04-2025/01).

### 2.2. Immunohistochemistry (IHC)

All sections received from the Multiple Sclerosis and Parkinson’s Tissue Bank were processed via immunohistochemical technique (IHC). IHC involves the use of monoclonal/polyclonal antibodies to establish tissue expression of an antigen of physiological and/or pathological interest [29]. Tissue sections underwent deparaffinization followed by heat-induced antigen retrieval (HIER), carried out according to the manufacturer’s instructions. This procedure utilized Coplin jars in combination with the EnVision FLEX Mini, High pH (Link)-K8023 kit (Agilent-Dako, Santa Clara, CA, USA).

To detect the TXNIP protein epitope, a primary mouse monoclonal anti-TXNIP antibody (clone 3A7.1, MABC605) was used at a 1:200 dilution (Merck KGaA, Darmstadt, Germany), prepared using the EnVision FLEX Antibody Diluent (K8006), which is included in the EnVision FLEX Mini Kit. Tissue sections were incubated with the primary antibody for approximately one hour.

The subsequent steps followed the manufacturer’s protocol for the EnVision FLEX Mini, High pH (Link)-K8023 kit. This included the use of EnVision FLEX Peroxidase-Blocking Reagent (SM801) and EnVision FLEX/HRP (SM802), both from Agilent-Dako (Santa Clara, CA, USA).

To visualize the antigen–antibody (Ag-Ab) complexes, a chromogenic solution was prepared by mixing 17 parts of the EnVision FLEX HRP Magenta Substrate Chromogen System (DM857) with 3 parts of the EnVision FLEX Substrate Buffer (DM843), according to the manufacturer’s guidelines (Agilent-Dako, Glostrup, Denmark).

After the immunohistochemical reactions, sections were counterstained with hematoxylin. Negative control reactions, as recommended by the antibody supplier, were included in all IHC procedures. Finally, tissue sections were dehydrated and coverslipped using xylene-based DPX mounting medium (BDH, Poole, UK).

### 2.3. Microscopic Analysis

All sections processed for immunohistochemistry were examined using an Olympus BX50 microscope (Olympus Italia S.r.l., Segrate, Italy), equipped with a digital camera for image capture. Images were acquired using the Olympus cellSens Standard software (version 1.18). Microscopic evaluations were conducted at magnifications ranging from 4× to 100×. The proportion of TXNIP-positive cells was independently quantified by two blinded co-authors (M.S. and F.A.S.), with no significant discrepancies observed between their assessments.

### 2.4. Statistical Analysis

In the histologic sections analyzed in this study (6 PD subjects and 6 healthy con-trols), a count of dopaminergic neurons within the SNpc that had positive and negative immunostaining was performed at a microscopic magnification of 20× and in 30 ocular fields. The values were then statistically processed by means of the Mann–Whitney U test (carried out with the commercially available software STATISTICA version 12, StatSoft Inc., Tulsa, OK, USA), which verifies the differences between two groups on a single ordinal variable without specific distribution and with non-parametric data [30,31].

## 3. Results

The principle of immunohistochemistry involves antigen–antibody binding. In this specific case, a monoclonal antibody against TXNIP binds to the N-terminal epitope of the TXNIP protein. This interaction subsequently allows for the visualization of its tissue expression through enzymatic methods, rendering the signal visible under a microscope.

**Figure 1:**
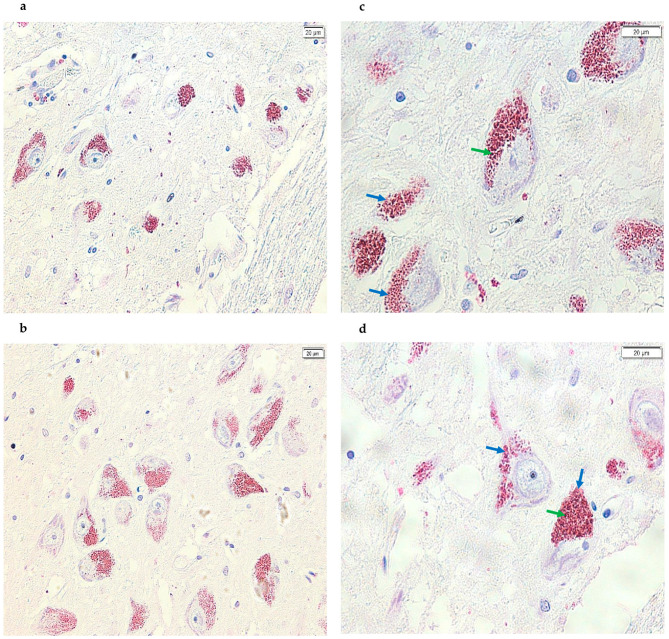
Images acquired using cellSens of SN sections from the post-mortem brains of healthy subjects. Specifically, using the TXNIP monoclonal antibody that binds the corresponding epitope, positive immunostaining (magenta) is detected at the cytoplasmic level, as well as in the same areas where NM was present (brown), resulting in a glossy staining. The signal is present and clearly visible in all samples analyzed (panels (**a**,**b**), 20× and panels (**c**,**d**), 40×). In addition, the green arrows point to the NM signal (brown), while the blue arrows point to the positive signal (magenta).

**Figure 2:**
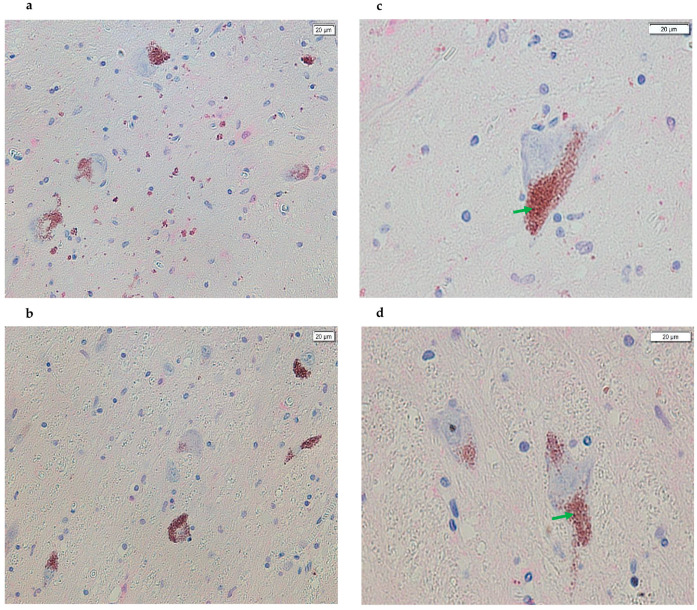
Images acquired using cellSens of SN sections from the post-mortem brains of subjects with PD. A difference in the positive (magenta) signal is evident in all images compared with the panels in Figure 1, indicating a reduction in or absence of magenta coloration in most NM granules and, therefore, a loss of antigen–antibody complex formation (panels (**a**,**b**), 20× and panels (**c**,**d**), 40×). In addition, the arrows in green point to the NM signal (brown).

**Figure 3:**
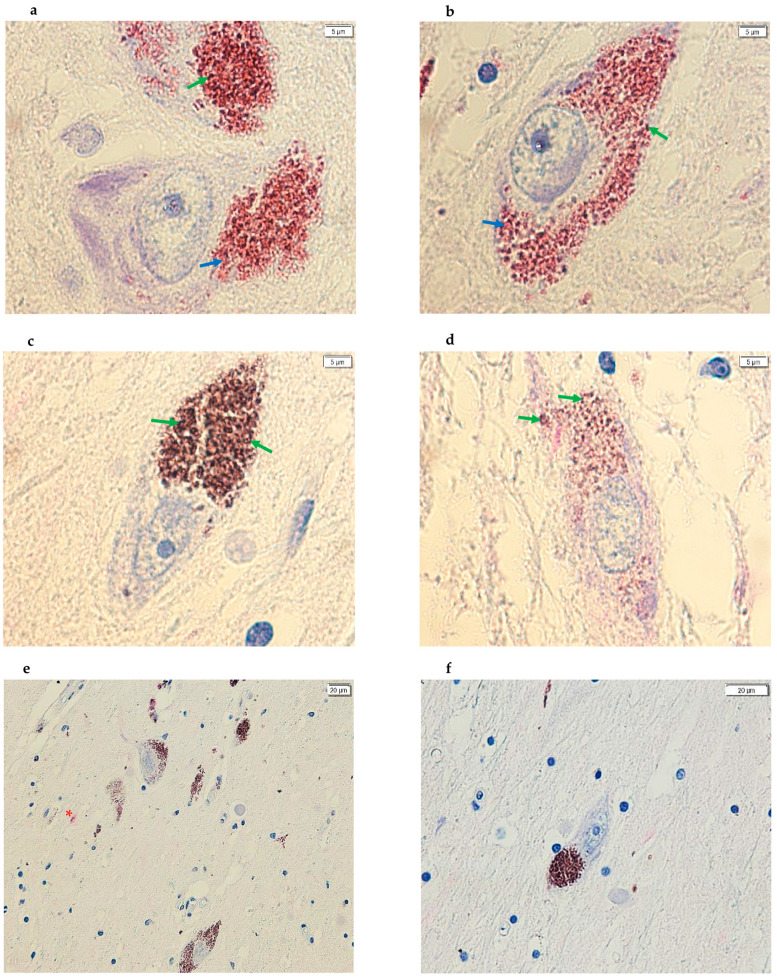
Images acquired using cellSens of SN sections from post-mortem brains. (**a**,**b**) The images show dopaminergic neurons, at 100× magnification, with positive immunostaining in proximity to the NM in CTRL subjects. (**c**,**d**) The images show dopaminergic neurons, at 100× magnification, with negative immunostaining in proximity to the NM in PD subjects. (**e**,**f**) The images show SNpc dopaminergic neurons in which the primary antibody was not added—a negative control; image (**e**) is from a CTRL subject while image (**f**) is from a PD subject. (panels (**a**–**d**), 100×; panel (**e**), 20× and panel (**f**), 40×). In addition, the arrows in green point to the NM signal (brown) while the arrows in blue point to the positive signal (magenta). Furthermore, the red asterisk in image (**e**) highlights a nonspecific pink coloration demonstrating that the magenta dye was introduced during the IHC experiment.

All control samples analyzed showed TXNIP protein expression. The SNpc is physiologically characterized by a high concentration of NM granules. In these control samples, positive immunostaining with a bright magenta, distinct from the typical brown color of NM, was evident at the location of these granules (Figure 1). This signal with positive immunostaining indicates specific antibody–antigen binding, confirming TXNIP protein expression in this area. In control samples, TXNIP protein expression seems to suggest a localization juxtaposed to NM and was not observed widely in the cytoplasm of the dopaminergic neurons identified (Figure 1). Conversely, within the dopaminergic neurons of the SNpc from PD subjects, TXNIP protein expression was observed to be significantly reduced or absent. When detectable, this protein expression appeared to be localized mainly near the NM. Similarly, the protein was not found to be diffusely expressed within the cytoplasm of identified dopaminergic neurons across all PD subject sections (Figure 2). Furthermore, Figure 3 also shows images of sections of dopaminergic neurons at the SNpc level, at 100× magnification, both in PD subjects and CTRL subjects (Figure 3, PD subjects c and d, while CTRL subjects a and b).

In addition, during the IHC protocol, two sections of post-mortem brain tissue from a subject with PD and a CTRL subject were also analyzed from the series of samples used in this pilot study, but without the addition of the primary antibody (Figure 3).

**Figure 4:**
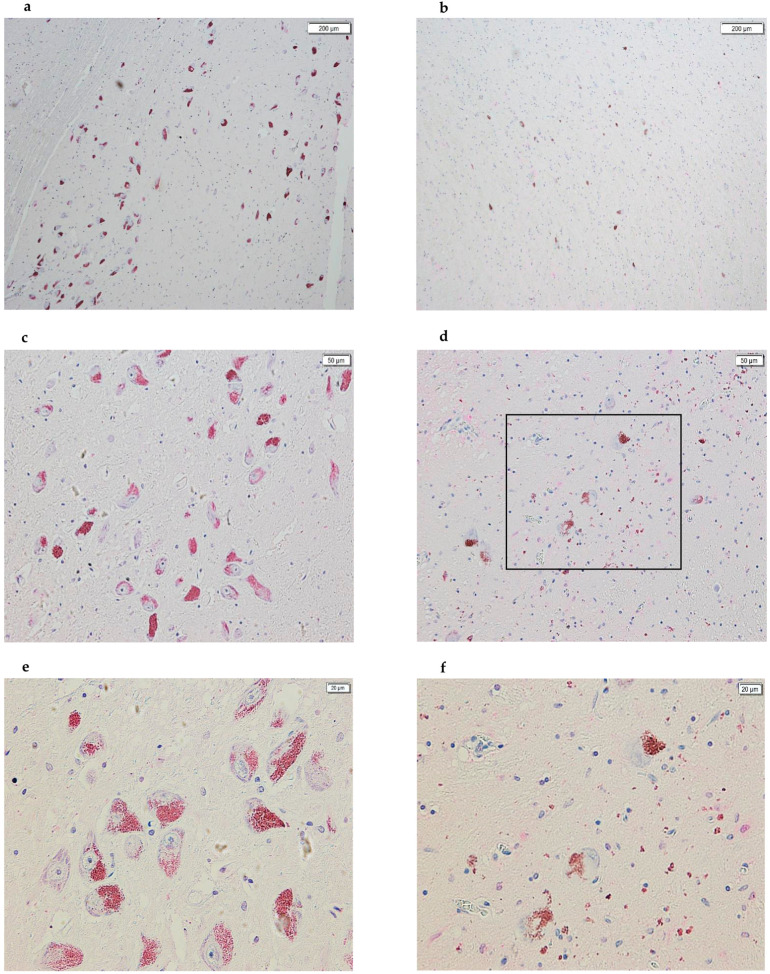
Images acquired using cellSens of SN sections from post-mortem brains. On the left, the panels of healthy subjects are shown, while on the right are the panels of subjects with PD. In all images, the following is evident: (1) a drastic decrease in dopaminergic neurons, part of the SNpc, between panels (**a**,**d**); (2) a drastic decrease in the amount of NM between panels (**b**,**e**); and, finally, (3) we hypothesize that all of the NM spots scattered in the PD panels are a consequence of the destruction of dopaminergic neurons and the release of the NM contained within them (panels (**a**,**b**), 4×; panels (**c**,**d**), 10×; panels (**e**,**f**), 20×). Panel (**f**) represents the enlargement of the part highlighted by the black box in panel (**d**).

Consistent with the existing literature, we also observed a drastic decrease in the number of NM-containing dopaminergic neurons in the SNpc of PD subjects compared to controls (Figure 4). Furthermore, we hypothesize that scattered NM spots observed in the tissue sections of subjects with PD are a consequence of dopaminergic neuron destruction and the subsequent release of their NM content.

Finally, no significant difference was found in the signal with positive immunostaining (TXNIP expression) in relation with age or sex within the samples studied. The obtained immunohistochemical data for TXNIP protein expression are consistent with the TXNIP gene expression findings from our previous transcriptomics study [26].

Table 2 reports the results of TXNIP immunostaining in dopaminergic neurons from both PD patients and healthy controls. For each subject, neuron counts with positive and negative immunostaining were assessed over 30 ocular fields at 20× magnification. In PD patients, the number of neurons with positive immunostaining ranged from 8 to 14, with a median value of 10.5 and an interquartile range (IQR) from 9 to 14. Conversely, the number of negatively immunostained neurons ranged from 75 to 115, with a median of 100 (IQR: 97–103). Conversely, all healthy controls exhibited high levels of TXNIP-positive staining, with values ranging from 496 to 539 and a median of 506 (IQR: 499–527), while none of the neurons in the control group showed negative immunostaining (all values = 0). The Mann–Whitney U test showed significant differences between the two groups. Namely, the number of TXNIP-positive neurons was significantly lower in PD patients compared to the controls (Z = −2.807, *p* = 0.005), while the number of TXNIP-negative neurons was significantly higher in the PD group (Z = 2.991, *p* = 0.0028). This indicates a marked reduction or even absence of TXNIP expression in the dopaminergic neurons of PD brains.

## 4. Discussion

This study suggests there is evidence for a significant alteration of the TXNIP expression and localization in the SNpc of brain sections from individuals with advanced PD. The main result seems to be a marked reduction in cytoplasmic TXNIP protein and, crucially, a loss of its close physical association with NM granules in PD samples, unlike healthy controls, where TXNIP was consistently detected in close proximity to NM. This protein-level alteration robustly aligns with our previous transcriptomic data, which indicated a significant downregulation of TXNIP mRNA in the SNpc of PD subjects [26,32,33,34]. It is interesting to note that other studies highlighted the role of TXNIP in other neurodegenerative diseases, such as AD, suggesting its causative role in the disease itself and a potential role as a therapeutic target [35,36,37].

The observed colocalization of TXNIP near the NM in healthy dopaminergic neurons may also provide a further contribution to the understanding of redox regulation in these cells. NM, an insoluble biopolymer that accumulates throughout life, plays a complex, often dual role in neuronal homeostasis. While it can act as a protective chelator of redox-active metals like iron, thereby mitigating oxidative stress, iron-laden NM can also become a source of reactive oxygen species, contributing to neuronal damage [20,38]. Given TXNIP’s established role as an inhibitor of thioredoxin, a key antioxidant enzyme, its strategic positioning with NM in healthy neurons suggests a sophisticated mechanism for modulating the local redox environment within these metabolically active, pigment-laden cells. This intricate relationship might contribute to maintaining a delicate oxidative balance under physiological conditions, critical for the long-term survival of dopaminergic neurons.

The precise molecular interactions between TXNIP and NM warrant further investigation, potentially involving direct protein–pigment binding or indirect scaffolding mechanisms. Indeed, we cannot exclude that dopaminergic neurons that express more TXNIP may be more protected from the neurodegenerative process underlying PD, although this needs to be addressed through mechanistic or functional studies, which are beyond the primary target of this pilot study. However, the data here obtained might be the starting point for further analyses specifically addressing these relevant aspects. In this context, it is worth noting that an immunohistochemical work has already been published [32] and this new study not only confirms previous transcriptomic data, but also demonstrates that a lower transcription of the mRNA corresponds to a lower translation of the protein in brain sections of advanced PD. Of course, this direct mRNA–protein correlation may not necessarily also be confirmed in subjects with previous (i.e., less severe) disease stages, but the proximity of the protein with NM may reinforce a mechanism that seems to correlate with the localization of many proteins within dopaminergic neurons.

The pronounced reduction in TXNIP protein and its dissociation from NM in late-stage PD (Braak LB stage 6) is highly indicative of the widespread loss of dopaminergic neurons and associated NM content, both hallmarks of advanced disease, which our study corroborated [39]. If TXNIP is indeed predominantly expressed within these specific neurons and functionally linked to NM, then the profound neuronal and pigment loss characteristics of late-stage PD would inevitably lead to a drastic decrease in detectable TXNIP. The presence of scattered NM remnants in PD tissue, hypothesized to be shed from degenerating neurons, further supports this notion, as these isolated NM aggregates exhibited negligible TXNIP staining. This suggests that TXNIP is either lost concurrently with neuronal integrity or its expression is severely diminished in compromised neurons. Indeed, the accumulation of NM has been linked to disruptions in cellular machinery and eventual cell death, and its presence is frequently associated with elevated inflammation in PD brains [20,22,40,41,42]. The release of NM into the extracellular space during neuronal degeneration is known to trigger neuroinflammatory responses, further contributing to the hostile microenvironment in the PD brain [43]. Our findings, showing a loss of TXNIP in this context, suggest that the neuroinflammatory processes might be exacerbated due to a disrupted cellular antioxidant defense system.

Notably, some authors highlighted the interaction between immune system dysregulation and neurodegenerative processes during the onset and development of PD, including alterations of the peripheral immune system and the involvement of microglia. Therefore, targeting microglial activation and immune dysfunction in individuals at risk of PD might represent a promising preventive measure and offer new therapeutic strategies for early intervention on this disease and other neurodegenerative disorders [44].

Our findings of significantly reduced TXNIP in late-stage PD present an interesting contrast with some existing research. For instance, Su et al. (2017) reported in a mouse model that TXNIP over-expression led to α-synuclein accumulation and dopaminergic neuron destruction by sensitizing cells to oxidative stress and impairing autophagy [9]. In 2020, Su et al. [45] used a mouse model of diabetes and observed an upregulation of TXNIP, which was associated with the downregulation of Parkinson’s disease-related genes and the regulation of mitophagy in PC12 cells. These apparent discrepancies highlight the possibility of a stage-dependent role for TXNIP in PD pathogenesis. It is plausible that an initial upregulation of TXNIP, as seen in some experimental models, might contribute to early pathological processes by exacerbating oxidative stress and protein aggregation, thereby initiating neuronal dysfunction. However, the significantly reduced TXNIP expression (both at the mRNA and protein levels) observed in our late-stage human PD cohort could represent an end-stage phenomenon. This reduction might be a consequence of widespread neuronal death; impaired cellular metabolism in the remaining, severely affected neurons; or a compensatory mechanism in response to chronic oxidative stress. This aligns with studies suggesting a biphasic or context-dependent role for TXNIP in various diseases [46,47]. Furthermore, the progressive loss of NM from dopaminergic neurons and its subsequent release into the surrounding tissue, as observed in PD progression [9], could directly contribute to the decreased detection of TXNIP protein if its stability or expression is intimately linked to the presence and integrity of NM granules. We can hypothesize a compensatory biological mechanism, although we cannot also exclude that the difference in species may have played a role, with murine models being used in the study of PD and in which the disease was induced [9,45,48]. The induction of the disease in murine models might lead to different considerations based on the histological evidence of the murine model compared to the human one.

The pathophysiology of PD is well-characterized by oxidative stress, mitochondrial dysfunction, and protein misfolding [49]. TXNIP’s established influence on redox homeostasis makes its dysregulation in PD particularly relevant. While a simplistic interpretation might suggest that reduced TXNIP would lead to less thioredoxin inhibition and, consequently, reduced oxidative stress, this notion is incongruent with the well-documented and pervasive oxidative burden in PD. Instead, the decreased TXNIP levels observed in our study likely reflect a complex cellular dysregulation. Compromised dopaminergic neurons in late-stage PD may be unable to maintain normal TXNIP expression due to metabolic exhaustion or severe cellular damage. Alternatively, the loss or alteration of critical cellular compartments, such as NM granules, which appear to be crucial for TXNIP function or localization in healthy neurons, could directly contribute to the observed reduction. This emphasizes that the loss of TXNIP in late-stage PD is not necessarily beneficial but rather an indicator of severe cellular compromise and a potential exacerbation of the oxidative imbalance. Our study further corroborates the established drastic decrease of dopaminergic neurons and NM in the SNpc of PD subjects, providing a robust anatomical context for our findings. The scattered NM observed in PD brain sections, likely remnants of degenerated neurons, aligns with the understanding of ongoing neuroinflammatory processes often found in regions with NM, further suggesting a complex interplay between neuronal degeneration, pigment release, and the local immune response.

Limitations of this study include the relatively small sample size, which, however, is common in post-mortem studies, and a sex imbalance between groups, although the intensity of positive immunostaining (magenta) did not significantly correlate with age or sex. Nevertheless, as known, sex differences may affect both gene expression [50] and clinical manifestations [51], thus influencing the prognostic trajectory and the treatment options of these patients [52].

The study focused on brain specimens from late-stage PD (i.e., Braak LB stage 6), which were those available at the time of the study, thus limiting the generalizability of the present results; however, it should be acknowledged that obtaining histological sections from non-advanced stages is rather difficult, as patients’ decease usually occurs during the advanced stages of the disease.

Future research should investigate TXNIP expression across different Braak stages to elucidate its dynamic role and explore the precise molecular interactions between TXNIP and NM and the functional consequences of TXNIP dysregulation in human dopaminergic neurons. As a study perspective, it is also interesting to evaluate the expression of the TXNIP protein in various stages of PD using more appropriate cellular models for PD; this would confirm the greater expression of the TXNIP protein at less severe Braak stages, thus opening relevant translational avenues for early detection and intervention.

## 5. Conclusions

In conclusion, this preliminary immunohistochemical study may suggest an alteration in TXNIP expression in the SNpc of brain sections from individuals with late-stage PD. Specifically, we observed a marked reduction in the staining of TXNIP proteins and a loss of its close juxtaposition with NM in the dopaminergic neurons of PD subjects compared to a clear expression of TXNIP related with NM found in healthy controls. These findings at the protein level are consistent with previous transcriptomic data showing *TXNIP* gene under-expression in the same cohort. While the precise implications of reduced TXNIP in advanced PD require further investigation, our results suggest that dysregulation of this key redox modulator may be a key feature of the complex neuropathology of PD in the SNpc, warranting further exploration of its role in neuronal vulnerability and disease progression.

## Figures and Tables

**Table 1:** All data from the six PD subjects and six CTRL subjects (age, sex, and clinical condition of the subject at the time of death).

PD Patients	Age	Sex	Clinical Diagnosis/Braak LB Stage
PD1	89	F	PD/LB findings, Braak LB stage 6, Limbic LBD
PD2	76	F	PD/LB findings, Braak LB stage 6, Amygdala-only LBD
PD3	79	M	PD/LB findings, Braak LB stage 6, Neocortical LBD
PD4	80	M	PD/LB findings, Braak LB stage 6, LBD
PD5	73	M	PD/LB findings, Braak LB stage 6, Neocortical LBD
PD6	80	M	PD/LB findings, Braak LB stage 6, Neocortical LBD
**Healthy Controls**	**Age**	**Sex**	**Clinical Diagnosis**
CTRL1	87	F	No neurological disorder
CTRL2	59	F	No neurological disorder
CTRL3	74	F	No neurological disorder
CTRL4	84	M	No neurological disorder
CTRL5	84	F	No neurological disorder
CTRL6	81	F	No neurological disorder

F = female; M = male; LBD = Lewy body disease; PD = Parkinson’s disease.

**Table 2:** Dopaminergic neuron counts with positive/negative immunostaining for TXNIP antibody performed in all sections. Evaluation of 30 ocular fields at 20× magnification.

PD Patients	Positive Immunostaining	Negative Immunostaining
PD1	10	98
PD2	11	75
PD3	14	97
PD4	9	102
PD5	14	115
PD6	8	103
**Median (Interquartile Range)**	10.5 (9–14)	100 (97–103)
**Healthy Controls**	**Positive Immunostaining**	**Negative Immunostaining**
CTRL1	502	0
CTRL2	527	0
CTRL3	496	0
CTRL4	539	0
CTRL5	510	0
CTRL6	499	0
**Median (Interquartile Range)**	506 (499–527)	0 (0–0)
**Mann-Whitney U test**	Z = −2.807, *p* = 0.005	Z = 2.991, *p* = 0.0028

**Note: *p*:** probability of obtaining a difference between the groups equal to or greater than that observed, assuming that there is no actual difference between the populations from which the samples originate. **Z:** used to determine statistical significance.

## Data Availability

Post-mortem human brain tissue sections were provided by the Multiple Sclerosis and Parkinson’s Tissue Bank located at Imperial College London, Hammersmith Hospital Campus, Du Cane Road (London W12 0NN, UK).
